# Salivary Hydrogen Sulfide Measured with a New Highly Sensitive Self-Immolative Coumarin-Based Fluorescent Probe

**DOI:** 10.3390/molecules23092241

**Published:** 2018-09-03

**Authors:** Ewelina Zaorska, Marek Konop, Ryszard Ostaszewski, Dominik Koszelewski, Marcin Ufnal

**Affiliations:** 1 Department of Experimental Physiology and Pathophysiology, Laboratory of the Centre for Preclinical Research, Medical University of Warsaw, 02-097 Warsaw, Poland; ewelina.zaorska@gmail.com (E.Z.); konopmarek@gmail.com (M.K.); 2 Institute of Organic Chemistry Polish Academy of Sciences, Kasprzaka 44/52, 01-224 Warsaw, Poland; ryszard.ostaszewski@icho.edu.pl

**Keywords:** fluorescent probe, hydrogen sulfide, azide, assay, Ellman’s Reagent, biological systems, saliva, halitosis

## Abstract

Ample evidence suggests that H_2_S is an important biological mediator, produced by endogenous enzymes and microbiota. So far, several techniques including colorimetric methods, electrochemical analysis and sulfide precipitation have been developed for H_2_S detection. These methods provide sensitive detection, however, they are destructive for tissues and require tedious sequences of preparation steps for the analyzed samples. Here, we report synthesis of a new fluorescent probe for H_2_S detection, 4-methyl-2-oxo-2*H*-chromen-7-yl 5-azidopentanoate (**1**). The design of **1** is based on combination of two strategies for H_2_S detection, i.e., reduction of an azido group to an amine in the presence of H_2_S and intramolecular lactamization. Finally, we measured salivary H_2_S concentration in healthy, 18–40-year-old volunteers immediately after obtaining specimens. The newly developed self-immolative coumarin-based fluorescence probe (C_15_H_15_N_3_O_4_) showed high sensitivity to H_2_S detection in both sodium phosphate buffer at physiological pH and in saliva. Salivary H_2_S concentration in healthy volunteers was within a range of 1.641–7.124 μM.

## 1. Introduction

Ample evidence shows that H_2_S plays a role of a mediator in many biological systems. For example, H_2_S has been found to contribute to the regulation of the circulatory system [1,2,3] nervous system [4,5], reproductive system [6,7,8] and energy balance [9,10]. In mammalian tissues, H_2_S is generated endogenously from cysteine and homocysteine. There are at least three enzymes that are responsible for converting sulfur-containing molecules into H_2_S: cystathionine β-synthase (CBS), cystathionine γ-lyase (CSE) and 3-mercaptopyruvate sulfurtransferase (MPST) [11,12]. Furthermore, H_2_S is generated in large amounts by microbiota, which is present in the intestines and in the oral cavity. On the one hand, microbiota-produced H_2_S seems to play a significant physiological role in cardiovascular and gastrointestinal systems [13,14,15,16,17,18,19]. On the other hand, the excessive bacterial production of H_2_S may cause medical complaints such as halitosis, a chronic bad breath condition [20,21].

Fast catabolism and low stability of H_2_S results in difficulties in the accurate analysis of H_2_S concentrations. Several methods have been traditionally employed for H_2_S detection, including colorimetric and electrochemical assays [22], gas chromatography and sulfide precipitation [23,24].

Most of these techniques require lengthy storing and/or complicated processing of analyzed sample. Therefore, new methods that will be useful for rapid and selective evaluation of H_2_S concentration in biological systems are highly desired. These requirements may be met by techniques employing fluorescent probes, which do not involve sophisticated sample processing and chemical treatment [25].

The goal of the study was to synthesize the probe that: (i) is facile to synthesize with an easy purification procedure (ii) acts fast (within seconds, considering real-time imaging of H_2_S-related biological processes), (iii) is chemically stable for long-term storage, (iv) shows a linear concentration–signal relationship within physiologically relevant H_2_S concentrations (v) is stable in aqueous solutions, especially in physiological pH of a body fluids. Finally, in order to confirm the usability of the designed 4-methyl-2-oxo-2*H*-chromen-7-yl 5-azidopentanoate (**1**) for the detection of H_2_S in biological samples, we aimed to establish salivary H_2_S concentration in healthy volunteers. 

## 2. Results and Discussion

Here, we have designed and synthesized a new fluorescent probe (C_13_H_11_N_3_O_4_, compound **1**) based on a coumarin scaffold. The operation of compound **1** is based on the azide group to amine group reduction mediated by H_2_S in combination with spontaneous intramolecular lactamization. The developed probe was characterized by ^1^H-NMR, ^13^C-NMR (The Supplementary Materials) Compound **1** showed dhe esired characteristics for a highly sensitive fluorescent probe for H_2_S detection. Compound **1** showed a good aqueous solubility and worked an an optimal pH ≈ 7.0, the pH of most of mammalian body fluids. Data analysis revealed a linear relationship between the fluorescence signal and the concentration of aqueous solutions of NaHS, a commonly used H_2_S donor*.* Finally, the compound **1** was synthesized from commercially available reagents in a straightforward procedure.

Currently, several methods for H_2_S detection are used, i.e., colorimetric and electrochemical assays and metal-induced sulfide precipitation. Despite the many advantages of the abovementioned methods their widespread use in biological systems is limited. This is due to the complex, multistep mechanisms of H_2_S detection, a slow response time, poor water solubility and poor stability in aqueous solutions of the reagents, and non-physiological pH of the reaction environment. Moreover, some of those methods require complicated sample processing steps and the destruction of cells or tissues. During the last years fluorescence-based probes have been attracting increasing interest as a method for H_2_S detection. The fluorescence-based assays for H_2_S detection offer high selectivity, sensitivity and biocompatibility, less invasiveness and enable real-time imaging [26,27,28]. Various fluorescence methods for H_2_S detection have been elegantly reviewed by Guo et al. [29]. The synthesis and design strategies of fluorescent probes are based on the use of specific chemical reactions and the use of several characteristic properties of H_2_S. The most commonly used strategy for designing fluorescent probes is the reduction of azide or nitro groups to amine groups [30,31,32,33,34]. Self-immolative probes based on coumarin were designed and synthesized by Han and co-workers [35]. Based on a similar strategy, Zhao and Song demonstrated a series of probes with para-azidobenzyl group attached to the 1,8-naphthalimide [36,37]. Other methods are based on unique dual nucleophilic reactions [25,38,39], high binding affinity towards copper ions [40,41,42] and a specific addition reaction to unsaturated double bond [43,44,45].

The evaluation of H_2_S concentration, or more precisely, free sulfhydryl group concentration may also be performed using Ellman’s reagent, i.e., 5,5′-dithiobis(2-nitrobenzoic acid), often referred to as DTNB [46,47,48]. However, the latter method requires alkaline conditions (pH 8.0) and the test absorbance response is obtained no sooner than after 15 min. Therefore, despite the progress in the field of detection methods, further development of highly sensitive and selective fluorescent probes for H_2_S detection is still needed to provide valuable information on the functions of H_2_S in physiological and pathological processes.

Saliva is a promising and increasingly used biological material for clinical investigations [49,50]. H_2_S in saliva may originate from its endogenous synthesis in tissues and from oral microbiota activity. The excessive concentration of H_2_S in the saliva is associated with halitosis [20,51,52].

In our study, using the newly synthesized probe we showed that the concentration of H_2_S in saliva of healthy 20–40-year-old humans is in the range between 1.641 and 7.124 μM (Table 1).

Our results are comparable to previously reported ones, however slightly higher [53,54,55]. Generally, the analysis of H_2_S is a tricky procedure because of the instability of H_2_S, its high volatility and rapid oxidation. This can lead to falsely elevated or decreased H_2_S concentrations. The most used methods for H_2_S detection are colorimetric assays (mainly the methylene blue method), high-performance liquid chromatography and gas chromatography [52]. However, there is much doubt about the reliability of abovementioned methods. Differences between the above methods and the fluorescence method using our probe include different duration of sample preparation and processing, and different measurement conditions. In our study, the collected saliva samples were tested instantly, whereas in other studies the samples were subjected to lengthy processing or storage. For example, in studies by Kaneshiro et al. and Ritz et al. saliva was collected by holding a cotton swab in the mouth for a few minutes [53,54]. Other methods require a chemical treatment with strong acid or base before analysis of H_2_S [36]. Some of those treatments can lead to falsely elevated or decreased H_2_S levels and/or cause irreversible destruction of the analyzed sample. For example, the methylene blue method uses acidic conditions (pH = 2) in which so-called acid labile sulfides (ALS) are formed. This contributes to falsely high H_2_S level readings. As pointed out by Siegel and Kanehira the methylene blue method may be disturbed by interference with other colored substances that interfere with the measurements, lowering the sensitivity of this method [55]. In contrast to the methylene blue method our measurements do not require any chemical pretreatment of the sample. Moreover, compound **1** works in an aqueous medium at pH = 7.4. Finally, the methylene blue method is a single point assay and does not monitor the H_2_S concentration in real time. There are also doubts about the repeatability of this method [56]. Another disadvantage of currently used methods is their long incubation periods, which are needed to achieve detection [57]. Ritz et al. analyzed H_2_S concentrations with the fluorescent probe SF4 which required up to 45 min of incubation with a chemosensor. Considering that H_2_S is a very volatile compound H_2_S concentrations may decrease significantly during such a long sample processing time.

## 3. Materials and Methods

### 3.1. Materials and Instruments 

Unless noted otherwise, reagents and solvents for synthesis were obtained from commercial suppliers and employed without further purification. Commercial reagents for quantitating sulfhydryl groups were purchased from Thermo Fisher Scientific (Waltham, MA, USA). Buffer reagents were purchased from Sigma Aldrich (Saint Louis, MO, USA) and were used without purification. All spectroscopic measurements were performed in 0.1 mM sodium phosphate buffer (pH 7.4) or 0.1 M sodium phosphate buffer (pH 8.0). Silica gel P60 (SiliCycle, Québec, QC, Canada) was used for column chromatography and SiliCycle 60 F254 silica gel (precoated sheets, 0.25 mm thick) was used for analytical thin layer chromatography and visualized by fluorescence quenching under UV light. UV/Vis spectra were recorded at ambient temperature using a U-1900 spectrophotometer (Hitachi, Chiyoda, Tokyo, Japan) and quartz cuvettes. Fluorescence spectra were recorded at ambient temperature in quartz cuvettes using a F7000 spectrofluorometer (Hitachi).

### 3.2. Synthesis and Sensing Mechanisms

**Figure 1:**
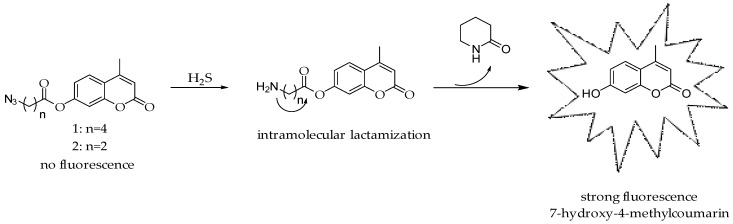
Mechanism of H_2_S detection for the self-immolative probe.

In the design of the probe for H_2_S we used 7-hydroxy-4-methylcoumarin as a fluorophore due to its good stability and desirable spectroscopic properties, such as large absorption extinction coefficients, sharp fluorescence emissions and excitation and emission in visible region [58,59]. The fluorescence of 7-hydroxy-4-methylcoumarin can be easily controlled by modification of hydroxyl group causing changes of physical and chemical properties and fluorescence quenching. Our probe operates by H_2_S-mediated reduction of azide group, which generates a primary amine, that can subsequently undergo spontaneous intramolecular lactamization to release 7-hydroxy-4-methyl-coumarin and piperidin-2-one (Figure 1). Our scientific concept is analogous to studies reported by Zadlo-Dobrowolska et al. for self-immolative carbonate-based probes [60]. Moreover, in comparison to other fluorogenic assays, self-immolative probes provide a more stable signal with higher signal to noise ratio.

**Figure 2:**
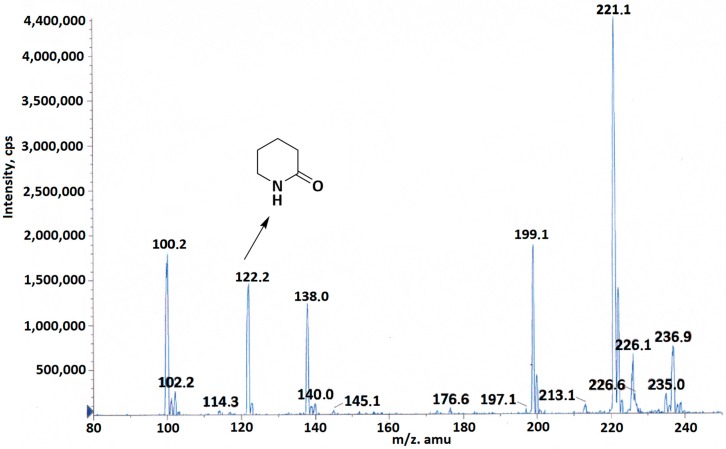
HRMS confirmed formation of piperidin-2-one in the reaction of the compound **1** with NaHS.

To confirm the proposed mechanism, the reaction solution was analyzed by high resolution mass spectrometry (HRMS) and NMR analysis. MS and NMR spectra confirmed formation of piperidin-2-one as a product of intramolecular lactamization. A major peak located at 122.2 corresponding to piperidin-2-one (C_5_H_9_NO, [M + Na]^+^: 122.07) was observed (Figure 2, the supplementary materials).

**Figure 3:**
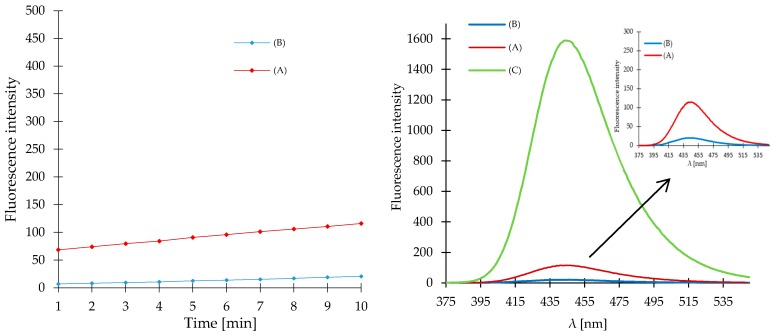
Time course experiment of compound **2** (0.1 mM, A) and **3** (0.1 mM, B) reacting with NaHS in sodium phosphate buffer (pH = 7.4) at room temperature. Time points represent time range from 1 to 10 min after addition of NaHS (0.1 mM). Fluorescence spectra of compound **1** (C), **2** (A) and **3** (B) were recorded for 10 min after addition of NaHS (0.1 mM).

In order to check the validity of the proposed mechanism we synthesized 4-methyl-2-oxo-2*H*-chromen-7-yl 3-azidopropanoate (**2**) and compared the results of *fluorometric* measurements for the 4-methyl-2-oxo-2*H*-chromen-7-yl 5-azidopentanoate (**1**) with the results obtained for compound **2** under the same reaction conditions. For compound **2** a much lower fluorescence response was recorded, which could be due to progressive autohydrolysis of compound **2** (Figure 3A). The hydrolytic decomposition of the compound **1** is further enhanced by close location of an electron-acceptor azide group in relation to the ester bond in the 7-position of 4-methylcoumarin. In addition, NMR and MS analysis of the assay solution was performed. In contrast to compound **1**, the 4-membered product of intramolecular lactamization was absent in the assay solution of compound **2** due to hydrolysis of the ester bond. 

To confirm the proposed mechanism of compound **1** in sensing H_2_S, 4-methyl-2-oxo-2*H*-chromen-7-yl propionate (**3**) was synthesized and tested in parallel under the same conditions. The analysis of reaction solution of compound **3** after addition of NaHS by fluorometry showed a minimal fluorescence enhancement (Figure 3B). In this case minimum fluorescence was caused the hydrolysis of the ester bond. The observed lower fluorescence enhancement of compound **3** in comparison to **2**, could result from the absence of the azide group in the structure of compound **3**. The different responses of 4-methyl-2-oxo-2*H*-chromen-7-yl propionate (**3**) and compound **1** highlighted a key role of the azide moiety for the H_2_S detection mechanism. NMR and MS analysis confirmed the absence of the lactamization product in the reaction mixture.

**Figure 4:**
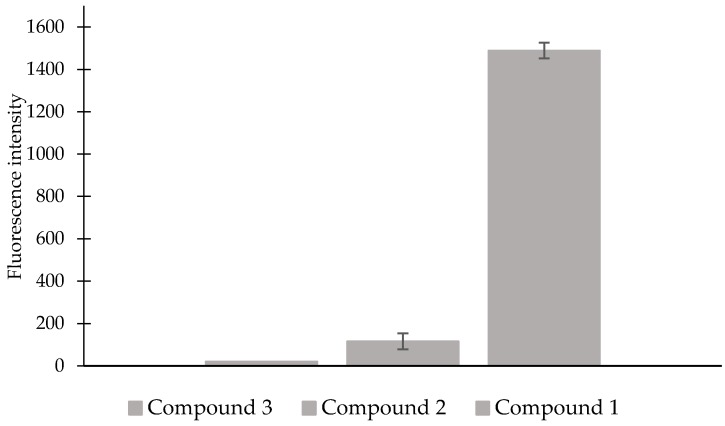
Comparison of fluorescence responses for the compound **1**, **2** and **3** (0.1 mM). Data were acquired at room temperature after addition of NaHS (0.1 mM) in sodium phosphate buffer (pH = 7.4, 20% CH_3_CN) with excitation at 365 nm. Means ± SE from three measurements of the fluorescence responses are presented.

To sum up, the compound **1** can be used for the determination of H_2_S levels. Furthermore, it is characterized by a high stability and the lack of susceptibility to autohydrolysis. We showed that the distance of the reaction site and thus the azide group as the electron-acceptor group from the fluorophore reduces the susceptibility to autohydrolysis (Figure 4). The obtained results confirm proposed mechanism of H_2_S detection for the compound **1**. Detection of H_2_S was achieved by the reduction of azide group mediated by H_2_S to amine group, then intramolecular lactamization with simultaneous release of highly fluorescent 7-hydroxy-4-methylcoumarin.

**Figure 5:**
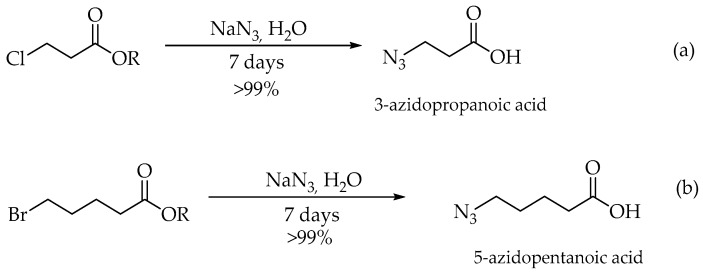
The synthesis of 3-azidopropanonic (**a**) acid and 5-azidopentanonic acid (**b**).

**Figure 6:**
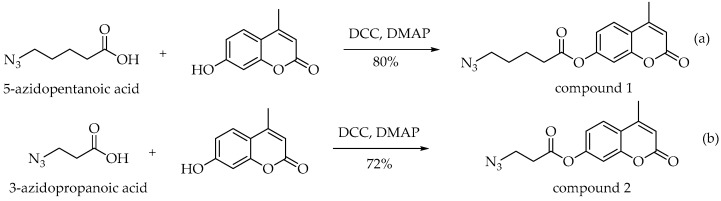
The synthesis of compounds **1** (**a**) and **2** (**b**).

Firstly, intermediates 3-azidopropanonic acid and 5-azidopentanonic acid were obtained by reacting suitable ester 3-chloropropionate (a) or ester 5-bromopentanoate (b) with sodium azide in H_2_O (Figure 5). The compound **1** and the compound **2** were synthesized from the corresponding commercially available fluorescent 7-hydroxy-4-methylcoumarin (Figure 6).

### 3.3. Synthesis of 3-Azidopropanoic Acid and 5-Azidopentanoic acid

A solution of sodium azide (4 equiv.) in 10 mL water was added dropwise into the ester 3-chloropropionate or ester 5-bromopentanoate (1 equiv.). The reaction mixture was stirred at room temperature for 7 days. After this time the resulting reaction mixture was acidified with solution of HCl (1 M). Then, the mixture was extracted with ethyl acetate for several times. The combined organic layers were dried over anhydrous MgSO_4_ followed by filtration and concentrated under reduced pressure. The obtained product was used for the next reaction without purification. Then, the azide-probe (**1**) and compound **2** was readily synthesized by esterification of 7-hydroxy-4-methylcoumarin with suitable azido acid in CH_2_Cl_2_, under a room temperature as shown in Figure 6.

**Figure 7:**
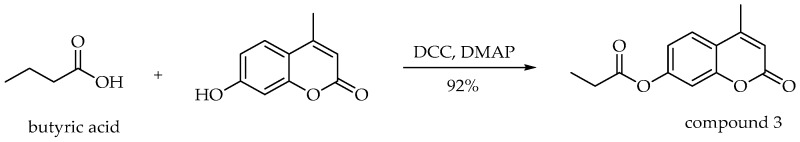
The synthesis of 4-methyl-2-oxo-2*H*-chromen-7-yl propionate (**3**).

In turn 4-methyl-2-oxo-2*H*-chromen-7-yl propionate (**3**) was synthesized from the corresponding commercially available 7-hydroxy-4-methylcoumarin with butyric acid in CH_2_Cl_2_, under room temperature as shown in Figure 7.

### 3.4. Synthesis of 4-Methyl-2-oxo-2H-chromen-7-yl 5-Azidopentanoate *(**1**)*

5-Azidopentanoic acid (1.2 equiv.), 7-hydroxy-4-methylcoumarin (1 equiv.), and a catalytic amount of *N,N*-dimethylpyridin-4-amine (DMAP) were dissolved in dry CH_2_Cl_2_ (15 mL). Then DCC (2 equiv.) was added. The reaction mixture was stirred at room temperature for 12 h. The reaction was monitored by thin layer chromatography (TLC). After the reaction was completed, the precipitate was filtered and washed several times with CH_2_Cl_2_. The filtrate was concentrated under reduced pressure. The crude product was purified by silica gel chromatography (ethyl acetate/n-hexane 3:7) to obtain the pure product as white solid (80% yield). ^1^H-NMR (400 MHz, CDCl_3_) δ 7.57 (d, *J* = 8.6 Hz, 1H), 7.13–6.93 (m, 2H), 6.22 (s, 1H), 3.33 (t, *J* = 6.6 Hz, 2H), 2.62 (t, *J* = 7.3 Hz, 2H), 2.39 (d, *J* = 1.1 Hz, 3H), 1.82 (dt, *J* = 12.0, 7.1 Hz, 1H), 1.76–1.64 (m, 1H); ^13^C-NMR (100 MHz, CDCl_3_) δ 170.94, 160.38, 154.14, 153.01, 153.01, 151.93, 125.41, 117.99, 117.80, 114.45, 110.31, 77.43, 77.11, 76.79, 50.98, 33.65, 28.17, 21.93, 18.64 ppm. Element. Anal. calcd. for C_15_H_15_N_3_O_4_: C 59.80, H 5.02, N 13.95; found C 59.72, H 4.94, N 13.84.

### 3.5. Synthesis of 4-Methyl-2-oxo-2H-chromen-7-yl 3-Azidopropanoate *(**2**)*

3-azidopropanoic acid (1.2 equiv.), 7-hydroxy-4-methylcoumarin (1 equiv.) and DMAP (a catalytic amount) were dissolved in dry CH_2_Cl_2_ (15 mL). Then DCC (2 equiv.) was added. The reaction mixture was stirred at room temperature for 12 h. The reaction was monitored by thin layer chromatography (TLC). After the reaction was completed, the precipitate was filtered and washed several times with CH_2_Cl_2_. The filtrate was concentrated under reduced pressure. The crude product was purified by silica gel chromatography (ethyl acetate/n-hexane 3:7) to obtain the pure product as white solid (72% yield). ^1^H-NMR (400 MHz, CDCl_3_) δ 7.59 (d, *J* = 8.6 Hz, 1H), 7.15–7.02 (m, 2H), 6.24 (d, *J* = 0.8 Hz, 1H), 3.69 (t, *J* = 6.4 Hz, 2H), 2.86 (t, *J* = 6.4 Hz, 2H), 2.41 (d, *J* = 0.9 Hz, 3H); ^13^C-NMR (100 MHz, CDCl_3_) δ 168.88, 160.32, 154.15, 152.69, 151.86, 125.49, 118.03, 117.89, 114.62, 110.30, 46.55, 34.17, 18.66 ppm. Element. Anal. calcd. for C_13_H_11_N_3_O_4_: C 57.14, H 4.06, N 15.38; found C 57.08, H 4.02, N 15.29. 

### 3.6. Synthesis of 4-Methyl-2-oxo-2H-chromen-7-yl Propionate *(**3**)*

Butyric acid (1.2 equiv.), 7-hydroxy-4-methylcoumarin (1 equiv.), and DMAP (a catalytic amount) were dissolved in dry CH_2_Cl_2_ (15 mL). Then DCC (2 equiv.) was added. The reaction mixture was stirred at room temperature for 12 h. The reaction was monitored by thin layer chromatography (TLC). After the reaction was completed, the precipitate was filtered and washed several times with CH_2_Cl_2_. The filtrate was concentrated under reduced pressure. The crude product was purified by silica gel chromatography (ethyl acetate/*n*-hexane 2:8) to obtain the pure product as a white solid (92% yield). ^1^H-NMR (400 MHz, CDCl_3_) δ 7.58 (d, *J* = 8.6 Hz, 1H), 7.31–6.75 (m, 2H), 6.22 (s, 1H), 2.62 (q, *J* = 7.5 Hz, 2H), 2.40 (d, *J* = 0.8 Hz, 3H), 1.26 (t, *J* = 7.5 Hz, 3H); ^13^C-NMR (100 MHz, CDCl_3_) δ 172.19, 160.40, 154.11, 153.20, 151.96, 125.37, 118.04, 117.67, 114.34, 110.30, 77.45, 77.13, 76.81, 27.69, 18.63, 8.88 ppm. Element. Anal. calcd. for C_13_H_12_O_4_: C 67.23, H 5.21; found C 67.19, H 5.17.

### 3.7. Characterization of the Fluorescence of Compound ***1***

**Figure 8:**
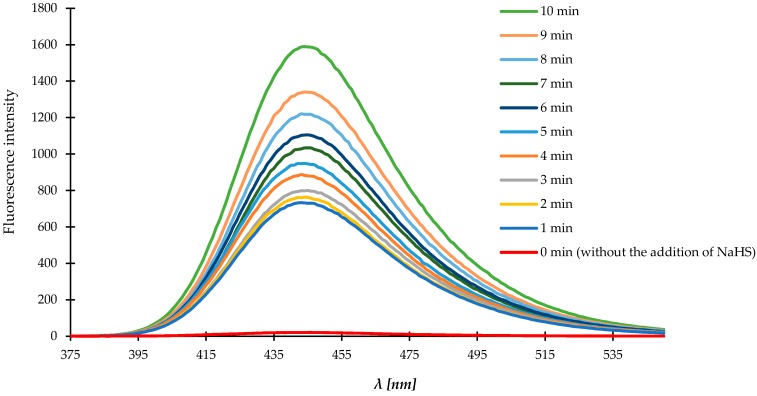
Fluorescence response of the self-immolative probe/compound **1** (0.1 mM) to 0.1 mM NaHS. Data were acquired at room temperature in sodium phosphate buffer (pH = 7.4) with excitation at 365 nm. Emission was collected in the time range 1–10 min after the addition of 0.1 mM NaHS. The spectrum at t = 0 min was acquired from a 0.1 mM solution of the compound **1** without the addition of NaHS.

First, we examined the optical properties of the probe/compound **1**. Compound **1** was non-fluorescent in sodium phosphate buffer containing 20% CH_3_CN at physiological pH 7.4. The sensing ability of H_2_S for the compound **1** was investigated using aqueous solutions of NaHS, a H_2_S donor. The solution was analyzed by fluorometry and spectra were recorded in selected time-points after the addition of NaHS. Upon addition of 0.1 mM NaHS, the solution of the compound **1** showed a strong fluorescence enhancement, as expected. A strong emission peak at 445 nm was detected when the reaction mixture was excited at 365 nm. The fluorescence intensity was dramatically increased due to the reduction of azide group to amine by H_2_S, intramolecular lactamization and the release of highly fluorescent 7-hydroxy-4-methylcoumarin. Within 10 min of reaction with NaHS (100 μM) the compound **1** generated an over 1000-fold fluorescence enhancement (Figure 8).

**Figure 9:**
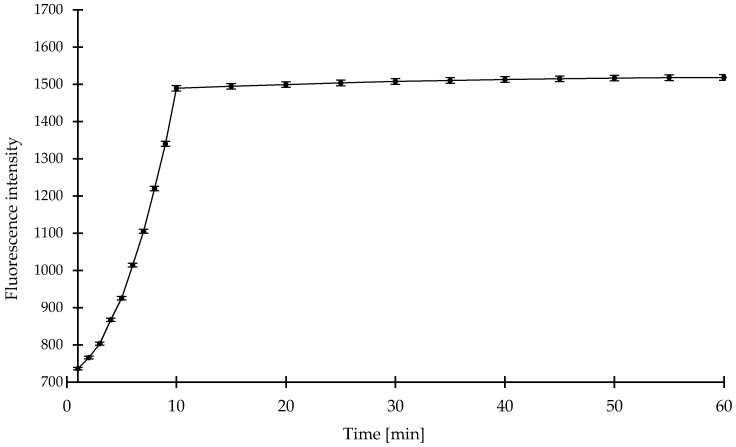
Time course experiment of the compound **1** (0.1 mM) reacting with NaHS (0.1 mM) in sodium phosphate buffer (pH = 7.4) at room temperature. Time points represent time range from 1 to 60 min after addition of NaHS (0.1 mM). Means ± SE from three measurements of the fluorescence responses are presented.

We examined the time courses of the fluorescence intensities of compound **1** in the presence of 100 μM NaHS. The time courses of the fluorescence intensities of the compound **1** (0.1 mM) in the presence of NaHS (0.1 mM) in sodium phosphate (pH = 7.4) buffered acetonitrile (20%, *v*/*v*) is displayed in Figure 9. The fluorescence signal increased rapidly at the beginning and reached steady state at around 10 min. When we extended reaction time to 60 min, the fluorescent intensity increased insignificantly, thus we chose 10 min as a test time.

**Figure 10:**
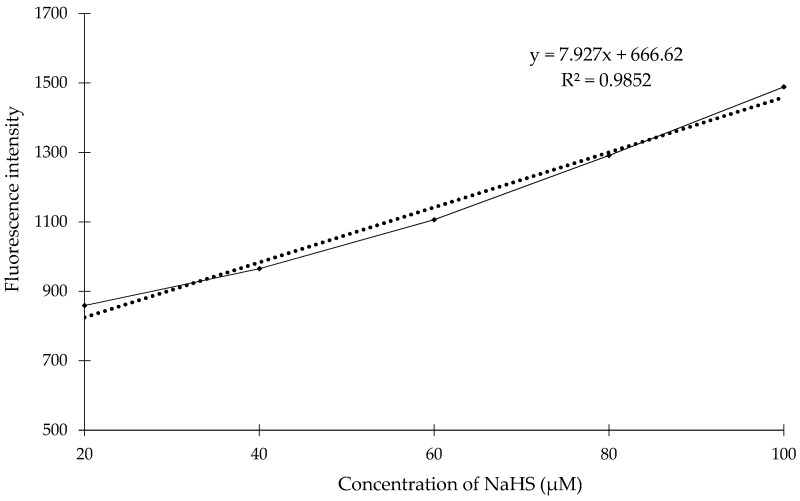
The correlation between fluorescence intensity and NaHS concentration determined using a fluorometer: the compound **1** (0.1 mM) with NaHS (20–100 μM) in sodium phosphate buffer (λ_ex_ = 365 nm) at room temperature. The points represent the mean fluorescence responses at 10 min after the addition of NaHS.

To evaluate the compound **1** for feasibility of quantitative determination of H_2_S concentration we examined the reactivity of the compound **1** in different concentrations of NaHS in sodium phosphate buffered acetonitrile (20% *v*/*v* CH_3_CN, pH = 7.4) at room temperature. NaHS (NaHS concentration from 20 μM up to 100 μM) was added to the test solution of the compound 1 (0.1 mM). As shown in Figure 10, we observed almost the linear relationship of fluorescence intensity of the compound **1** against varying concentrations of NaHS. The regression analyses was: *F_Ex_*_/*Em*_ (365/445 nm) = 7.927[H_2_S] + 666.62 with *R*^2^ = 0.985 in 10 min of incubation with NaHS.

### 3.8. Quantification of H_2_S Concentration Using Ellman’s Reagent (5,5-Dithiobis(2-Nitrobenzoic Acid) and the Developed Probe

We determined H_2_S levels in 20 μM up to 100 μM NaHS solution using DTNB method and our probe. DTNB assay was performed according to the protocol provided by the manufacturer (catalog number: 22,582, Thermo Fisher Scientific, Waltham, MA, USA).

**Figure 11:**
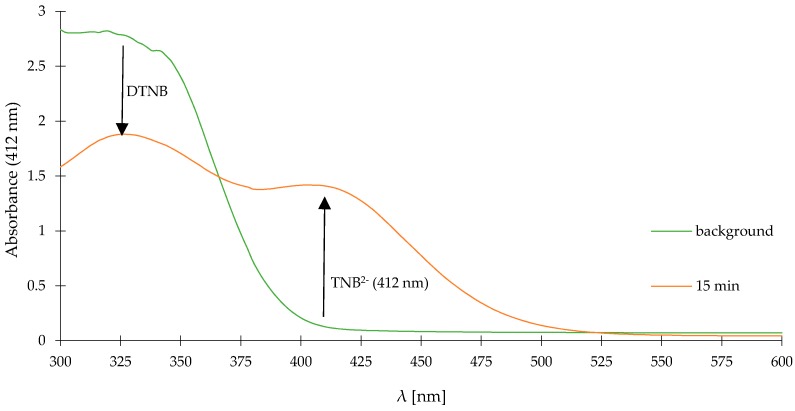
Time-dependent UV-vis absorption spectra of SH-free DTNB solution (green line-background) and its mixture with NaHS (0.1 mM, orange line) in the reaction buffer (pH 8.0) at room temperature. The absorbance at 412 nm was recorded 15 min after addition of NaHS.

Figure 11 shows time-dependent UV-vis absorption spectra of SH-free DTNB solution (green line-background) and its mixture with NaHS (0.1 mM, orange line). After 15 min incubation, the effect reaction of DTNB with NaHS on absorption spectra was observed as gain of TNB^2−^ (2-nitro-5-thiobenzoate anion) and a loss of DTNB, respectively. We chose 15 min as a test time, because after this time we did not observe any increase in the intensity of absorbance, 15 min is also the incubation time which is required for the measurement procedure recommended by the manufacturer.

**Figure 12:**
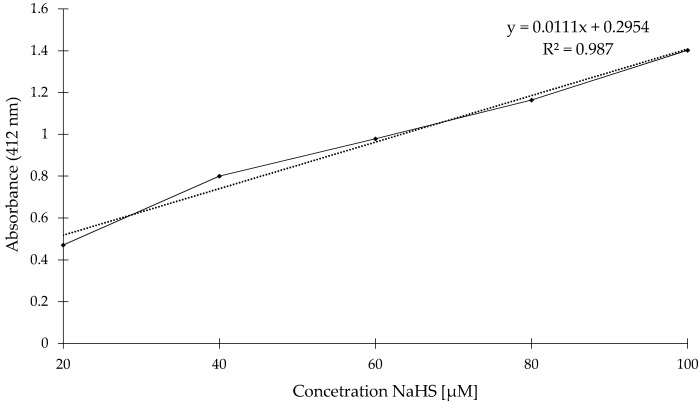
The correlation between absorbance and NaHS concentration determined by the DTNB assay in sodium phosphate buffer (pH 8.0) at room temperature. The absorbance at 412 nm was recorded for 15 min after addition of various concentrations of NaHS from 20 to 100 μm. Values of absorbance are given as means obtained from 3 measurements. Background values were subtracted from the sample values.

To determine relationship between changes of absorbance and concentration of NaHS, we recorded the absorbance in different concentrations of NaHS aqueous solution. Concentration of NaHS from 20 μM up to 100 μM, were used. As shown in Figure 12, we observed an increase in the absorbance along with increasing NaHS concentration.

Table 2 summarizes the results of H_2_S detection (aqueous solution of NaHS, a H_2_S donor) which have been obtained by Ellman’s test and by fluorescence method using our probe. In Table 2 are shown the results of H_2_S detection (aqueous solution of NaHS, a H_2_S donor) which have been obtained by Ellman’s test and by fluorescence method using our probe.

### 3.9. H_2_S Detection in Saliva

**Figure 13:**
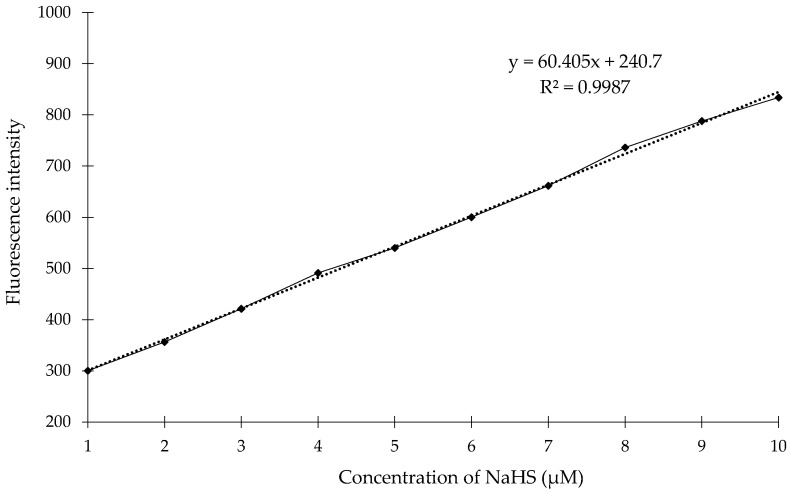
The correlation between fluorescence intensity and NaHS concentration determined using a fluorometer: the compound **1** (0.1 mM) with NaHS (1–10 μM) in sodium phosphate buffer (λ_ex_ = 365 nm) at room temperature. The points represent the mean fluorescence responses at 10 min after the addition of NaHS.

To determine whether the novel compound **1** can be used for the determination of H_2_S concentrations in a biological sample we performed competition experiments in 15 samples of saliva. Additionally, we plotted the calibration curve for the compound 1 in range concentrations of NaHS from 1 μM up to 10 μM. As shown in Figure 13, we observed almost the linear relationship of fluorescence intensity of the compound **1** in this range concentration of NaHS. The regression analyses were: *F_Ex_*_/*Em*_ (365/445 nm) = 60.405[H_2_S] + 240.7 with *R*^2^ = 0.9987. The obtained fluorescence data for saliva samples were converted into H_2_S concentrations by means of a calibration curve. The obtained results measured by fluorescence method with the compound **1** for 15 samples of saliva are presented in Table 1. 

### 3.10. General Procedure for H_2_S Detection by the Fluorescence Method

NaHS solutions with appropriate concentrations were prepared using sodium phosphate buffer as a solvent. For the assay, the various volumes of NaHS solution were added respectively to solution of 1, 2 and 3 in sodium phosphate buffer containing 20% CH_3_CN (pH = 7.4). The total volume of the solution being measured was 2000 μL. The final concentration of the compound **1** was 0.1 mM, while various concentration of NaHS were added (in range from 1 to 10 μM and in range from 20 to 100 μM). The fluorescence response was monitored over time. Emission spectra were collected between 350 nm and 550 nm with λ_ex_ = 365 nm. Time points represent time range from 1 to 10 min after addition of NaHS. The spectrum at t = 0 min was acquired from a 0.1 mM solution of the compound **1** without NaHS. Fluorescence data and obtained linear calibration curves were used to calculate the reaction rate of NaHS with the compound **1** and concentrations of H_2_S. 

### 3.11. General Procedure for H_2_S Detection Using the Ellman’s Test

Procedure was carried out quantitating sulfhydryl groups according to the manual attached by Thermo Fisher Scientific (Catalog number: 22,582). The procedure for the quantification of sulfhydryl groups was performed according to the manual attached by Thermo Fisher Scientific (Catalog number: 22,582).

### 3.12. General Procedure for H_2_S Detection in Saliva Using Compound ***1***

In these experiments an appropriate sample of saliva (0.4 mL) were added to solution of the compound **1** in the in sodium phosphate buffer containing 20% CH_3_CN (pH = 7.4). The final concentration of the compound 1 was 0.1 mM. The total volume of the solution being measured was 2000 μL. The fluorescence response of the compound **1** was monitored over time with λ_ex_ = 365 nm and λ_em_ = 445 nm. The spectrum at t = 0 min was acquired from a solution of the compound **1** without saliva. Fluorescence data were converted into H_2_S concentrations in sample of saliva by means of a calibration curve.

### 3.13. Collection of Saliva Samples

The study was performed in compliance with the ethical guidelines of the 1975 Declaration of Helsinki and was approved by the Bioethics Committee of the Medical University of Warsaw (approval no. KB/138/2018). Informed consent was obtained in every case. Samples were obtained from 15 adult volunteers. Demographics and clinical data of the study subjects are listed in Table 3. Inclusion criteria were as follows: healthy, 18–40 years-old, male and female. Exclusion criteria were as follows: chronic general diseases, acute general diseases, current dental problems, halitosis, treatment with any drugs or dietary supplement including probiotics during the last month before the study. Subjects brushed teeth and did not drink and eat for 90 min before saliva collection. Samples were collected directly to Eppendorf tubes after short exposition of subjects to the smell of lemon.

## 4. Conclusions

We synthesized a new fluorescent, self-immolative probe for H_2_S detection in biological fluids. The design of compound **1** was based on the combination of two strategies for H_2_S detection, i.e., reduction of an azido group to an amine in the presence of H_2_S and spontaneous intramolecular lactamization. The compound **1** showed several characteristics that are desirable for evaluation of H_2_S concentration in biological systems and human body fluids, including straightforward synthesis, stability, reactivity in aqueous media at physiological pH and fast response time. Finally, we measured salivary H_2_S concentration in healthy, 18–40-year-old volunteers immediately after obtaining specimens. Salivary H_2_S concentration in healthy humans was within a range of 1.641–7.124 μM. 

## Figures and Tables

**Table 1:** Salivary H_2_S concentration in healthy volunteers.

Samples of Saliva (n = 15)			
H_2_S concentration [μM]	Range	Mean	SE
	1.641–7.124	3.424	±0.547

**Table 2:** Comparison of detection methods for H_2_S.

Concentration of NaHS [μM]	Ellman’s Test (μM) ^a^		Fluorescence Method Using the Compound 1 (μM) ^b^	
	C H_2_S[μM]	V [μM s^−1^]	C H_2_S[μM]	V [μM s^−1^]
20	15.82	0.018	17.00	0.028
40	36.91	0.041	38.15	0.064
60	57.63	0.064	58.10	0.097
80	78.25	0.087	78.60	0.13
100	94.52	0.105	97.23	0.16

^a^ Conditions: 10 mM Ellman’s Reagent Solution in sodium phosphate buffer (pH 8.0), room temperature, the absorbance at 412 nm was recorded 15 min after addition of NaHS. ^b^ Conditions: 0.1 mM the fluorogenic probe in sodium phosphate buffer (pH 7.4), room temperature, the fluorescence intensity was recorded 10 min after the addition of NaHS.

**Table 3:** Demographics and clinical data of the study subjects and H_2_S levels in the saliva samples obtained using fluorescence method with the compound **1**.

**Participant Demographics and Physical Characteristics**.			
Healthy volunteers (n)	15		
Age (years)	Mean	SE (standard error)	Range
	28	1.6	18–40
Sex (m/f)	7/8		
**Ethnicity:**			
Caucasian	100%		
Other	-		

## Supplementary Materials

The Supplementary Materials are available online.

## Abbreviations

ALS	Acid Labile Sulfide
CDCl_3_	Deuterated chloroform
CH_2_Cl_2_	Dichloromethane
CH_3_CN	Acetonitrile
CLSS	ChemiLuminescent Sulfide Sensors
^13^C-NMR	Carbon-13 nuclear magnetic resonance
CuS	Copper sulfide
CTAB	Cetyltrimethylammonium bromide
DCC	*N*,*N*′-Dicyclohexylcarbodiimide
DMAP	*N*,*N*-Dimethylpyridin-4-amine
DTNB	5,5′-Dithiobis(2-nitrobenzoic acid)
HCl	Hydrochloric acid
^1^H-NMR	Proton nuclear magnetic resonance
HRMS	High Resolution Mass Spectrometry
H_2_S	Hydrogen sulfide
MgSO_4_	Magnesium sulfate
mM	Millimolar concentration
MS	Mass Spectrometry
NaHS	Sodium hydrosulfide
R^2^	Coefficient of determination
SE	Standard Error
SF	Sulfur fluride
TLC	Thin layer chromatography
TNB^2−^	2-Nitro-5-thiobenzoate anion
UV-vis	Ultraviolet–visible spectroscopy
λ_ex_	Excitation wavelength
λ_em_	Emission wavelength
