# Supplementary figures and images for: Salivary Hydrogen Sulfide Measured with a New Highly Sensitive Self-Immolative Coumarin-Based Fluorescent Probe
Source: Molecules. 2018 Sep 3;23(9):2241. doi: 10.3390/molecules23092241 (PMC6225104; doi:10.3390/molecules23092241)

7.58  
7.56  
7.07  
7.07  
7.05  
7.04  
7.02  
7.02

6.22  
6.22

3.35  
3.33  
3.31

2.64  
2.62  
2.60  
2.39  
2.39

1.85  
1.83  
1.83  
1.81  
1.72  
1.70  
1.70  
1.68

4-methyl-2-oxo-2H-chromen-7-yl 5-azidopentanoate (1)

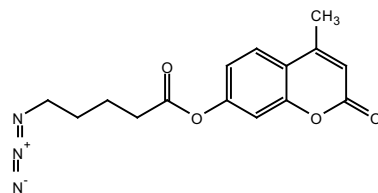

I (d)  
7.57

H (m)  
7.04

G (d)  
6.22

F (t)  
3.33

E (t)  
2.62

D (d)  
2.39

C (dt)  
1.82

B (m)  
1.70

A (s)  
1.22

1.73

3.41

1.66

3.49

3.50

5.25

3.87

3.65

1.00

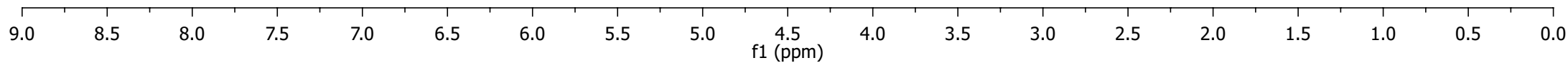

Supplement: Supplementary file 1 [file molecules-23-02241-s001.zip › 1H NMR compound 1.pdf]

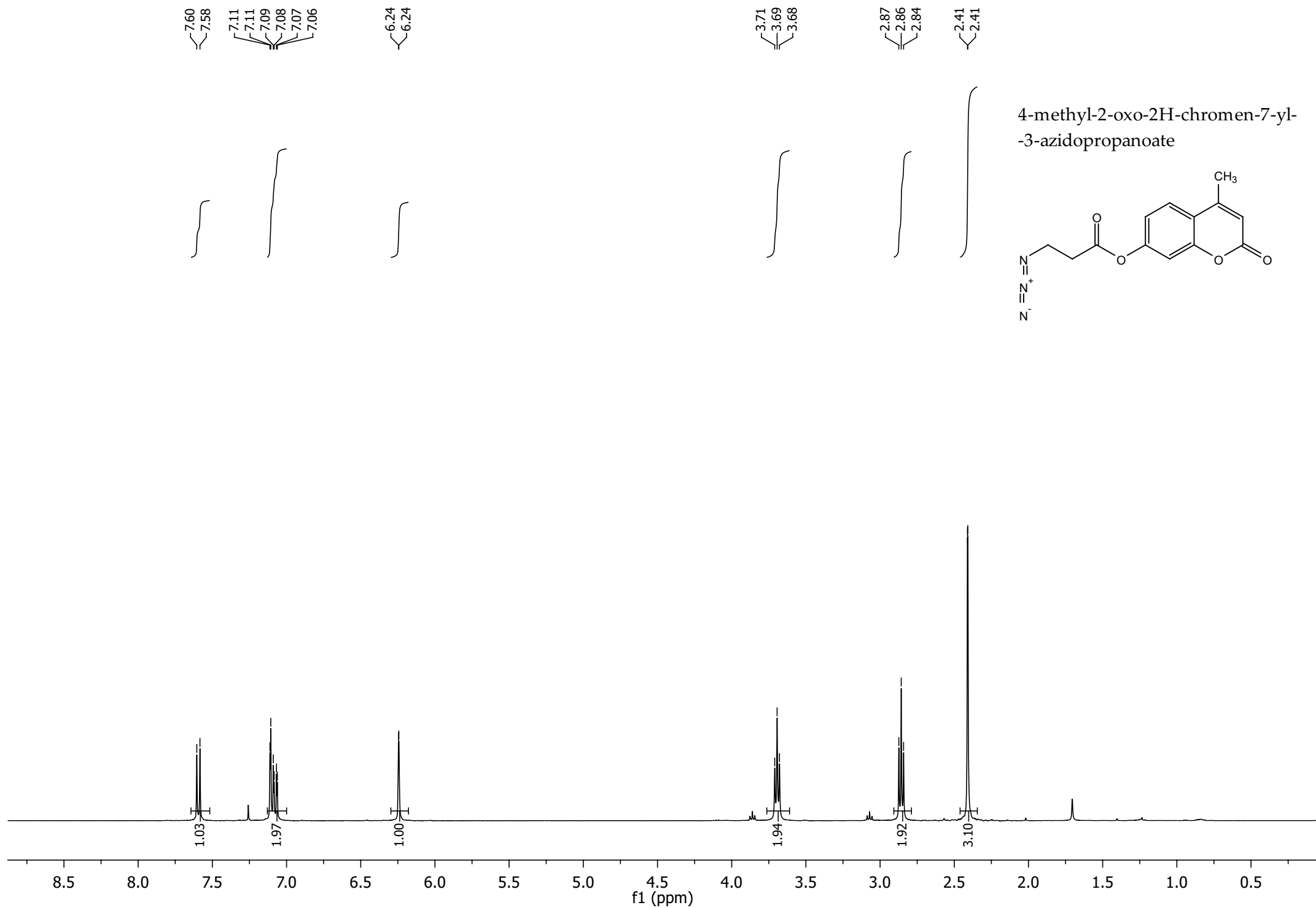

Supplement: Supplementary file 1 [file molecules-23-02241-s001.zip › 1H NMR compound 2.pdf]

4-methyl-2-oxo-2H-chromen-7-yl propionate (3)

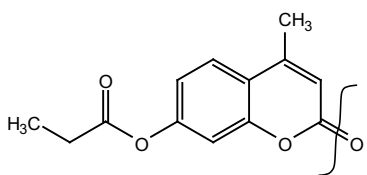

7.59  
7.57  
7.08  
7.07  
7.06  
7.06  
7.04  
7.03

6.22

2.64  
2.62  
2.61  
2.59  
2.40  
2.40

1.28  
1.26  
1.24

F (d)  
7.58

E (m)  
7.06

D (s)  
6.22

C (q)  
2.62

B (d)  
2.40

A (t)  
1.26

0.33

0.65

0.32

0.68

1.02

1.00

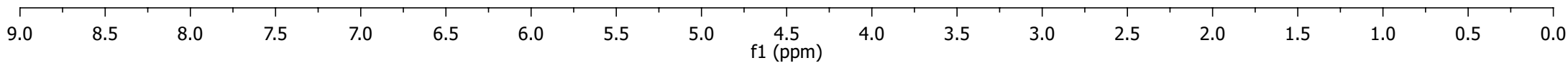

Supplement: Supplementary file 1 [file molecules-23-02241-s001.zip › 1H NMR compound 3.pdf]

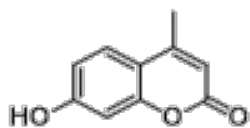

7.42  
7.37

6.80  
6.79  
6.77

6.04  
6.04

2.35  
2.34

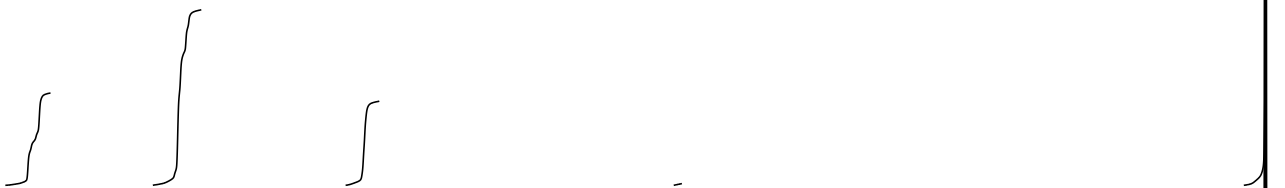

1.10

2.09

1.00

0.02

3.30

8.5 8.0 7.5 7.0 6.5 6.0 5.5 5.0 4.5 4.0 3.5 3.0 2.5 2.0 1.5 1.0 0.5

f1 (ppm)

Supplement: Supplementary file 1 [file molecules-23-02241-s001.zip › 7-Hydroxy-4-methylcoumarin-1H NMR.pdf]

4-methyl-2-oxo-2H-chromen-7-yl 5-azidopentanoate (1)

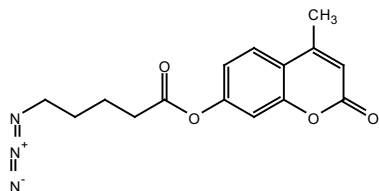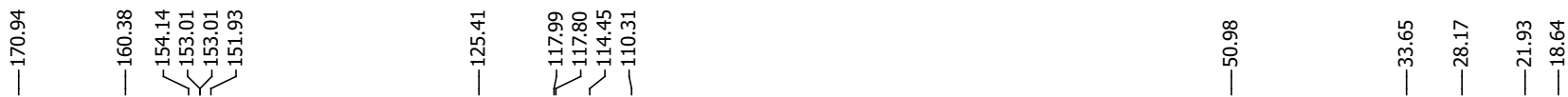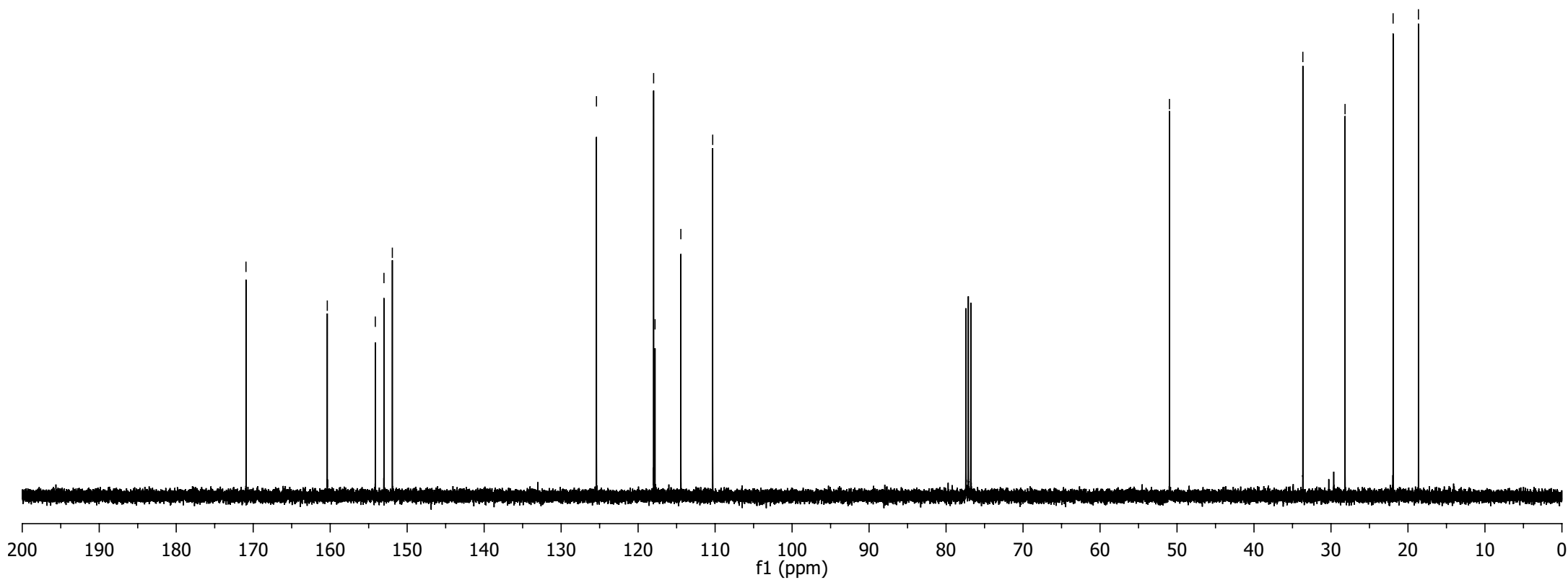

Supplement: Supplementary file 1 [file molecules-23-02241-s001.zip › 13 C NMR compound 1.pdf]

4-methyl-2-oxo-2H-chromen-7-yl-  
-3-azidopropanoate

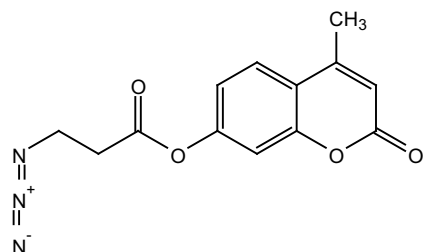

—168.88  
—160.32  
—154.15  
—152.69  
—151.86

—125.49  
—118.03  
—117.89  
—114.62  
—110.30

—46.55

—34.17

—18.66

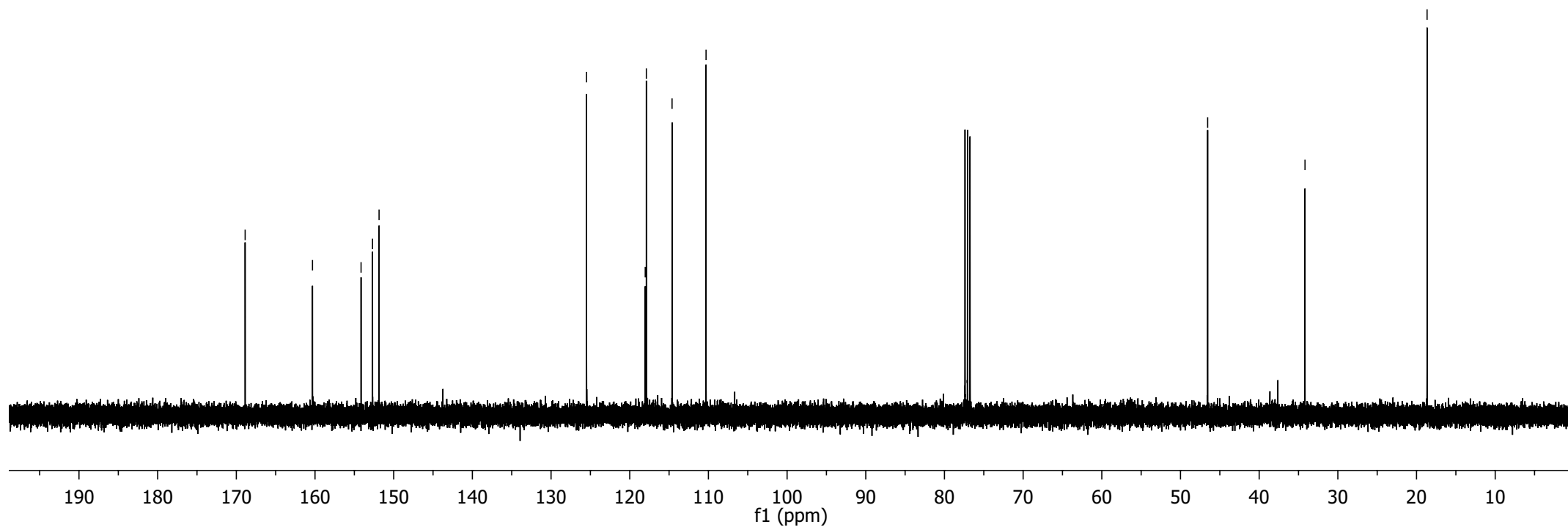

Supplement: Supplementary file 1 [file molecules-23-02241-s001.zip › 13 C NMR compound 2.pdf]

4-methyl-2-oxo-2H-chromen-7-yl propionate (3)

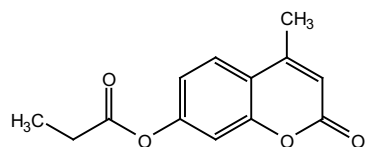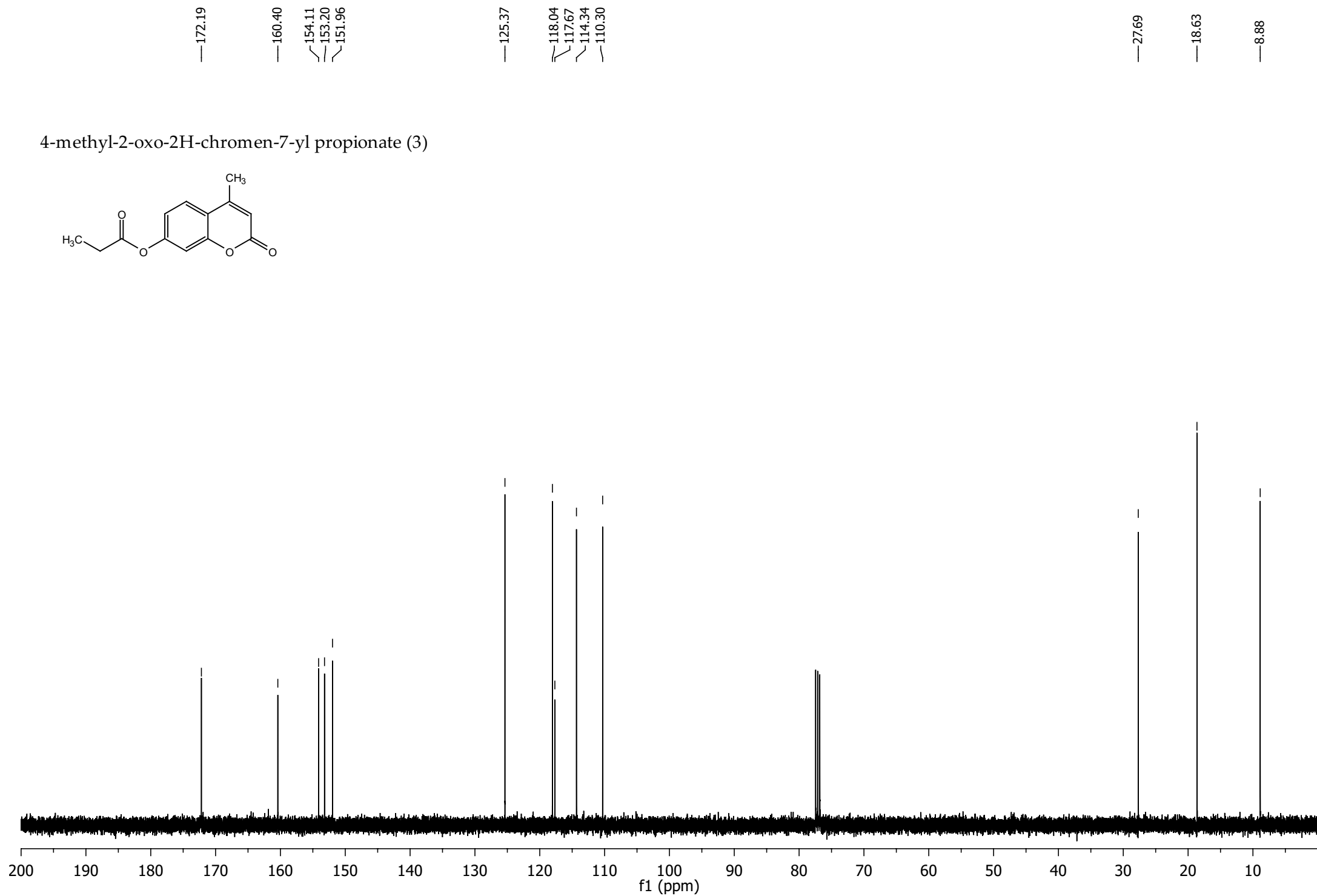

Supplement: Supplementary file 1 [file molecules-23-02241-s001.zip › 13C NMR compound 3.pdf]

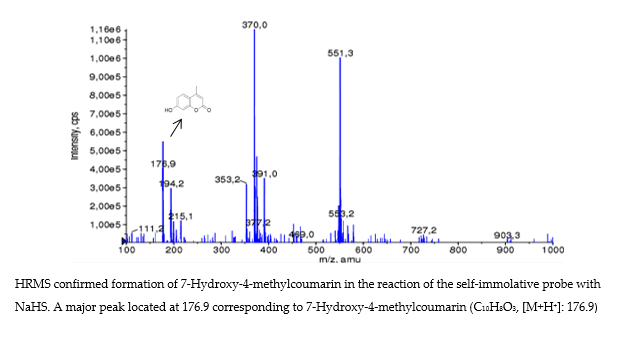

Supplement: Supplementary file 1 [file molecules-23-02241-s001.zip › HRMS - 7-Hydroxy-4-methylcoumarin.png]
